# Supplementary material for: Desmin intermediate filaments and tubulin detyrosination stabilize growing microtubules in the cardiomyocyte
Source: Basic Res Cardiol. 2022 Nov 3;117(1):53. doi: 10.1007/s00395-022-00962-3 (PMC9633452; doi:10.1007/s00395-022-00962-3)
Supplement: Supplementary file 1 — Supplementary file1 (DOCX 2456 KB) [file 395_2022_962_MOESM1_ESM.docx]

**Supplemental Figures**


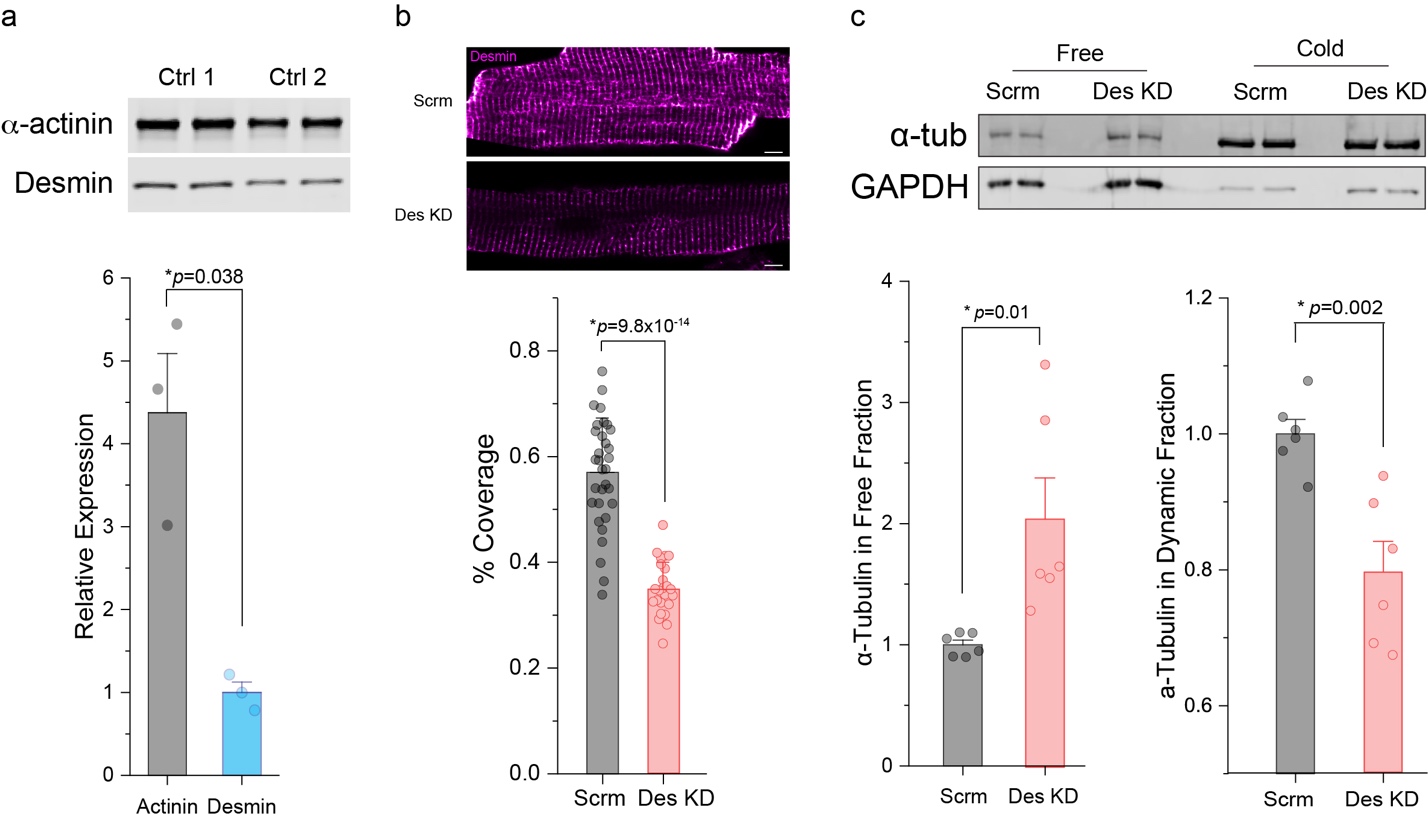


**Supplemental Fig. 1 (a)** Representative western blot images **(top)** and quantification **(bottom)** of α-actinin and desmin using whole cell lysates from adult rat cardiomyocytes; the blots were immunostained using the same α-actinin and desmin antibodies and concentration as used in Figure 1e (N=3 rats, n=3 averaged-values from 2 WB technical lanes per rat). **(b)** Representative immunofluorescent images **(top)** and quantification of coverage area **(bottom)** for desmin in scramble shRNA (Scrm) and Desmin Knock-Down shRNA (Des KD) adult rat cardiomyocytes (N=3 rats, n=~10 cells per rat). **(c)** Representative western blot **(top)** and quantification **(bottom)** of α-tubulin and GAPDH in free and cold-sensitive fractions from rat cardiomyocytes infected with adenovirus containing Scrm and Des KD (N=3 rats, n=6 WB technical lanes). The bar represents mean ±1 SEM; statistical significance is determined with Two-sample Student’s T-test.

**
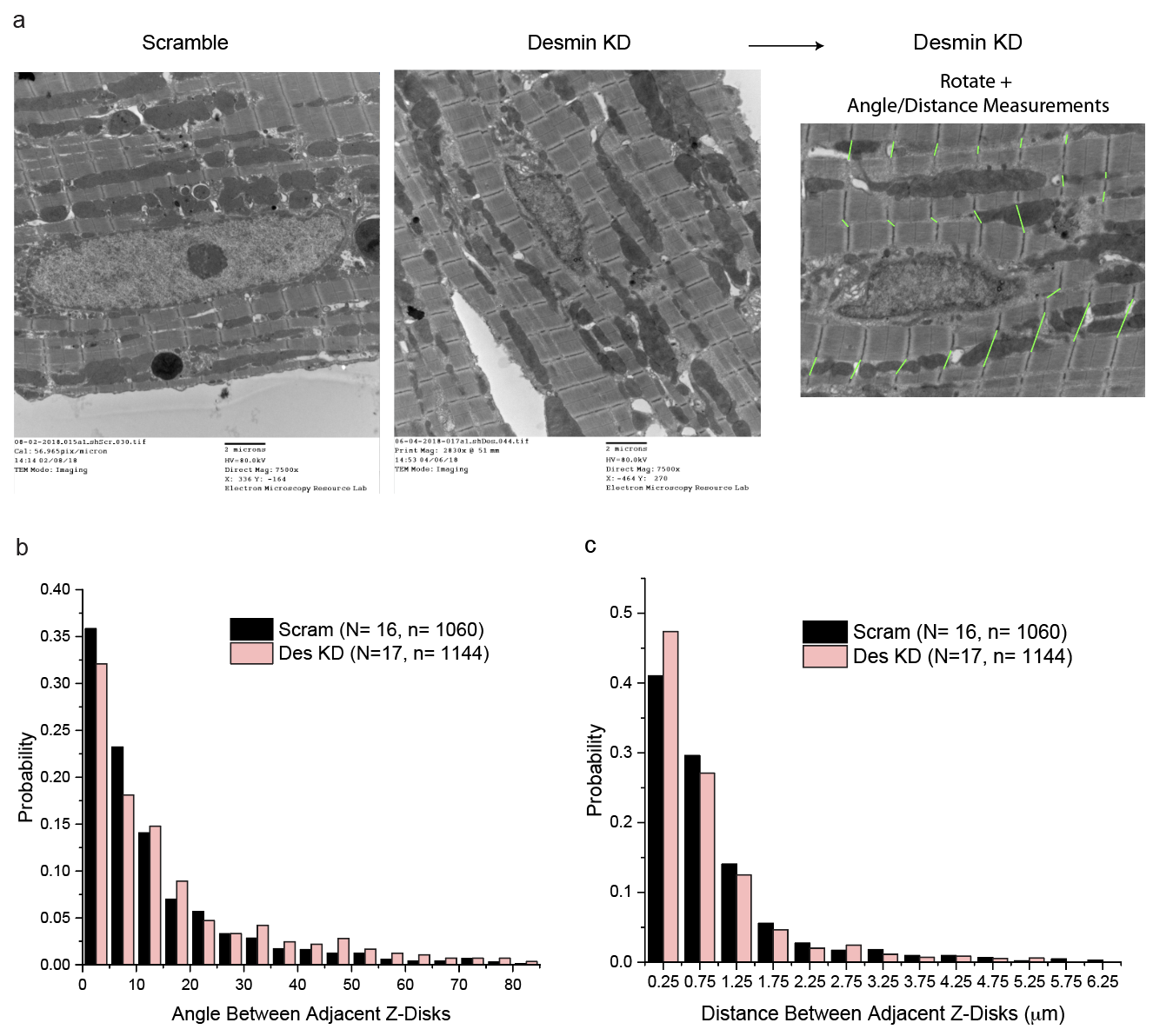
**

**Supplemental Fig. 2 (a)** Representative electron microscopy images of isolated rat cardiomyocytes with or without desmin knockdown. **(b)** Histograms of angle and **(c)** distance between adjacent Z-discs with or without desmin knockdown (N=cells, n=events).

**
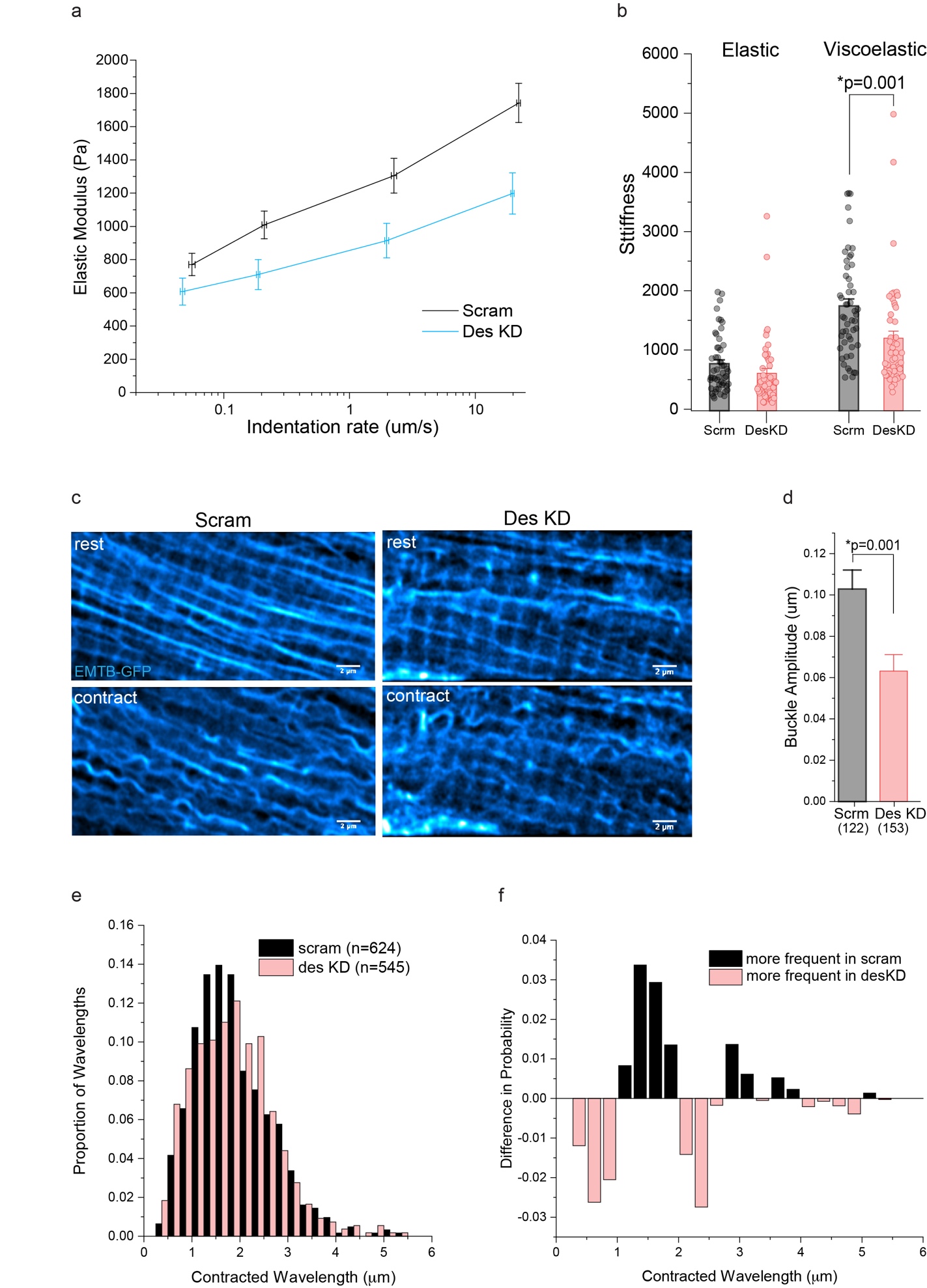
**

**Supplemental Fig. 3 (a)** Nanoindentation measurement of cardiomyocyte viscoelasticity displayed as stiffness (elastic modulus) as a function of probe indentation velocity with or without desmin KD. **(b)** Quantification of rate-dependent (viscoelastic) and independent (elastic) stiffness; the height of bar graphs denotes mean and error bar ±1 SE. **(c)** Representative images of microtubules in a cardiomyocyte at rest **(top)** and the peak of contraction **(bottom)** with or without desmin KD. **(d)** Quantification of microtubule buckle amplitude; the bar represents mean ± 1 SEM (n=cells). **(e)** Histogram of the wavelength distribution of individual microtubule buckles. **(f)** distribution difference (Scram – des KD from E). Statistical significance was determined using Two-sample Student’s T-test.

**
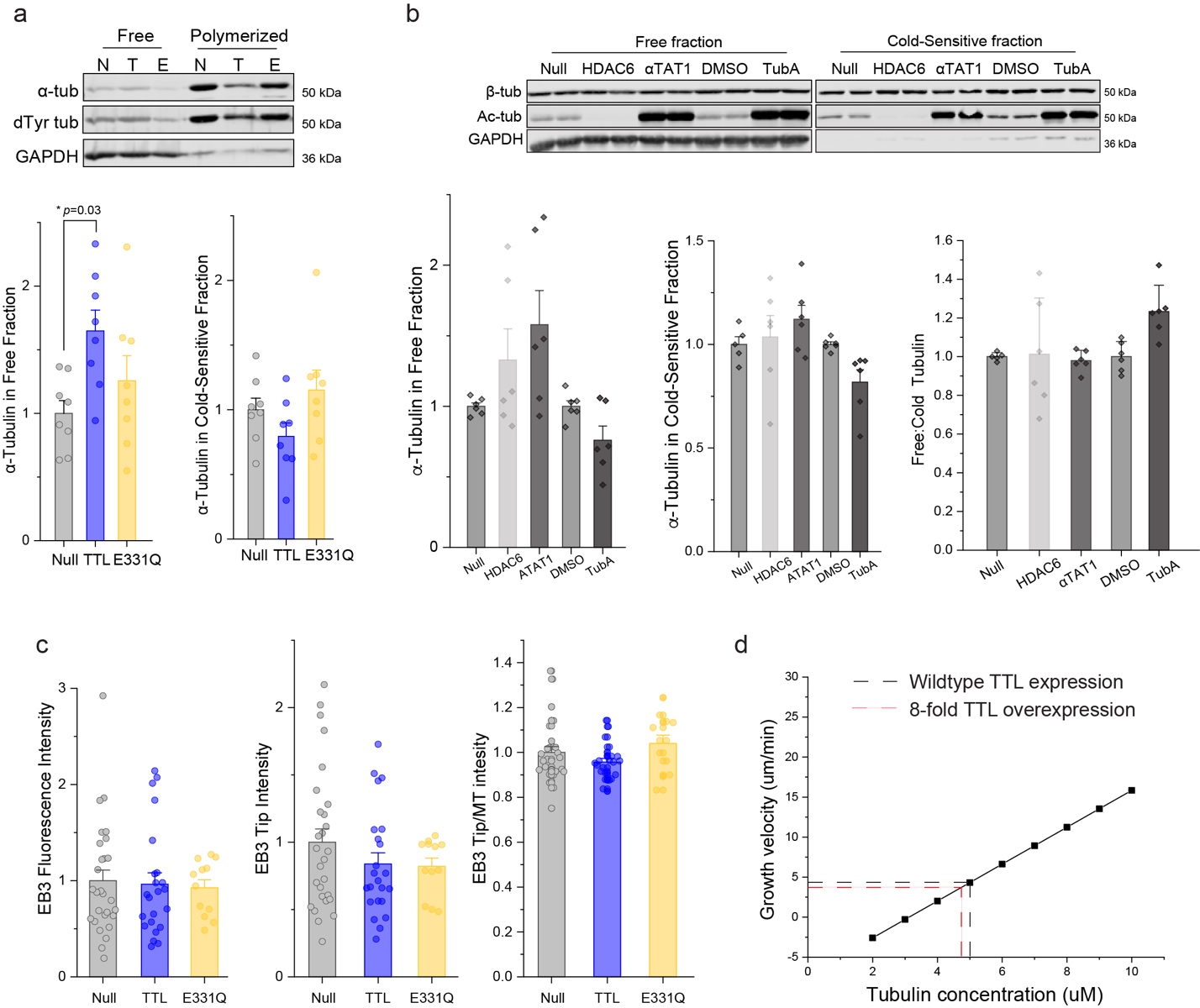
**

**Supplemental Fig. 4 (a)** Representative western blot **(top)** and quantification **(bottom)** of a-tubulin in free and cold-sensitive fractions from adult rat cardiomyocytes infected with null, TTL, or E331Q adenovirus (N=4 rats, n=8 WB technical lanes). **(b)** Representative western blot **(top)** and quantification **(bottom)** of β-tubulin and acetyl tubulin in free and cold-sensitive fractions from adult rat cardiomyocytes infected with either null, HDAC, or ATAT1 adenovirus, or treated with DMSO or TubA (N=3 rats, n=6 WB technical lanes). **(c)** Background **(left)** EB3-GFP fluorescence intensity, EB3 tip fluorescence intensity **(middle)**, and the ratio of EB3 tip fluorescence intensity to microtubule EB3 intensity **(right)** in adult rat cardiomyocytes co-infected with EB3-GFP and either Null, TTL, or E331Q adenoviruses. The bar represents mean ± 1 SEM; statistical significance was determined using one-way ANOVA with post hoc test. **(d)** To reduce levels of detyrosination in this study we relied on overexpression of TTL, which is known to associate with the free α/β tubulin heterodimer in a 1:1 complex with a K_d_ of 1 μM[38]. Due to the nature of this interaction, TTL overexpression can decrease the amount of “polymerization-competent” tubulin, leading to a decrease in *in vitro* microtubule polymerization[38]. The relationship between free tubulin concentration and microtubule polymerization is described as a simple 1D model below, where ν_g_ is the velocity of microtubule growth, *d* is the dimer length (assumed to be 8 nm), k_on_ is the tubulin dimer association constant per protofilament (assumed to be 4.8 μM^-1^s^-1^ )[24], k_off_ is the tubulin dissociation rate constant per protofilament (assumed to be 15s^-1^ )[24], and [Tb] is the concentration of free tubulin[4].

$$\nu_{g}=d\left( k_{on}\left[ Tb \right]- k_{off} \right).$$

Solving the equation of the line at a physiologic tubulin concentration of 5μM provides a theoretical value of 4.32 μm s^-1^ for growth velocity. Using the K_d_ value for TTL-tubulin interaction at a tubulin concentration of 5μM suggests that an 8-fold overexpression of TTL[9] will produce 0.2 μM of TTL-tubulin complex. If we assume that tubulin bound to TTL cannot be polymerized, then only 4.8 μM of free tubulin is polymerization-competent at any time. This change in polymerization-competent tubulin would lead to a ~10% decrease in growth velocity. We did not observe an effect of E331Q on microtubule growth kinetics or on event frequency suggesting that the local tubulin concentration at the microtubule plus-tip is unaffected by the sequestration activity of TTL. The bar represents mean ± 1SEM; statistical significance for (a) and (c) was determined using one-way ANOVA with post hoc test and for (b) was using Two-sample Student’s T-test.


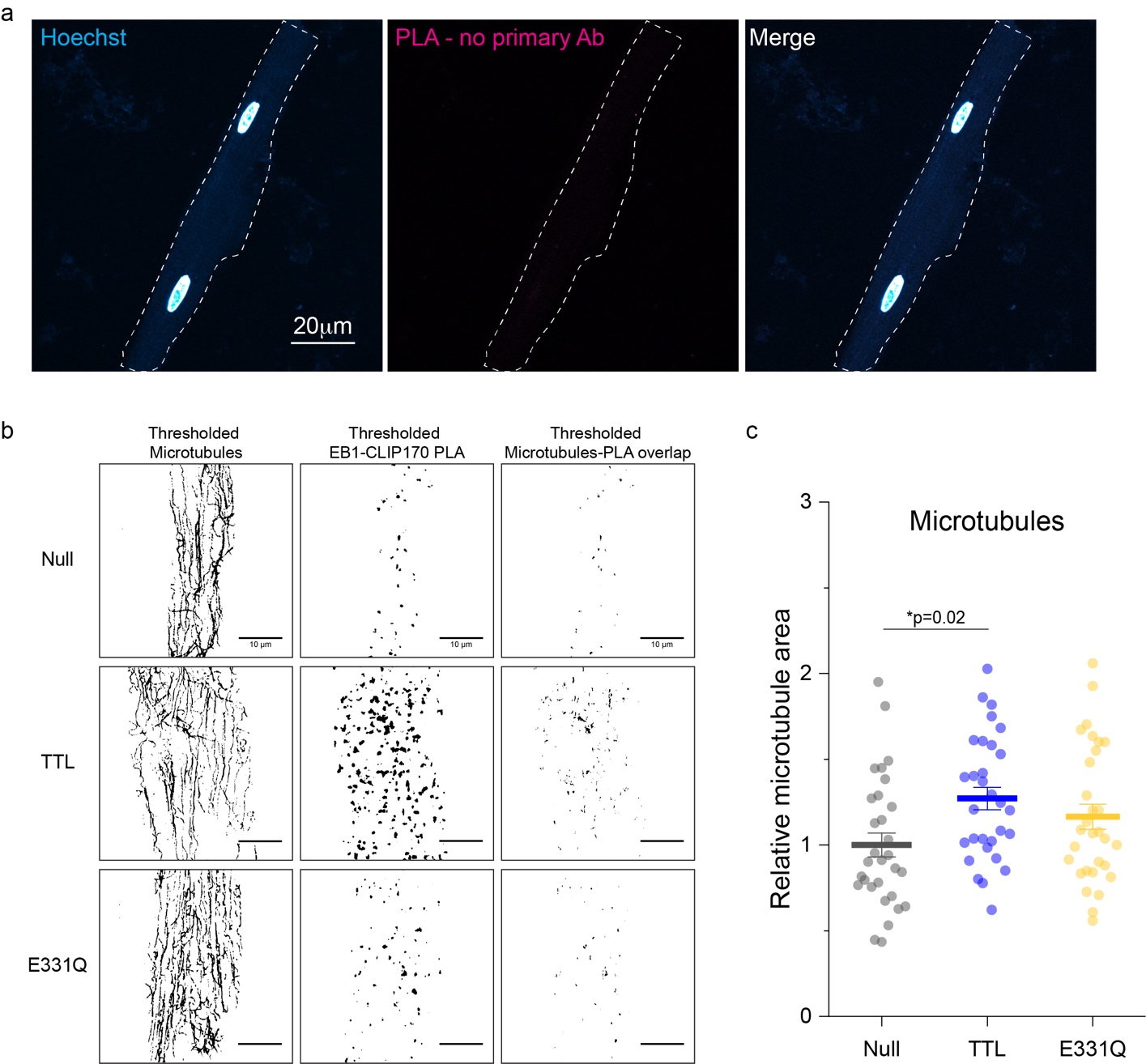


**Supplemental Fig. 5 (a)** Representative negative control immunofluorescent images after PLA in adult rat cardiomyocytes without EB1 or CLIP170 primary antibodies. **(b)** Representative thresholded immunofluorescent images used in the analysis and quantification of EB1-CLIP170 in Figure 5d. **(c)** Quantification of microtubule area in adult rat cardiomyocytes treated with Null, TTL, or E331Q adenovirus for 48h (N=3 rats, n=10 cells per rat). Bar

represents mean ±1 SEM; statistical significance determined with one-way ANOVA with post hoc test.

**
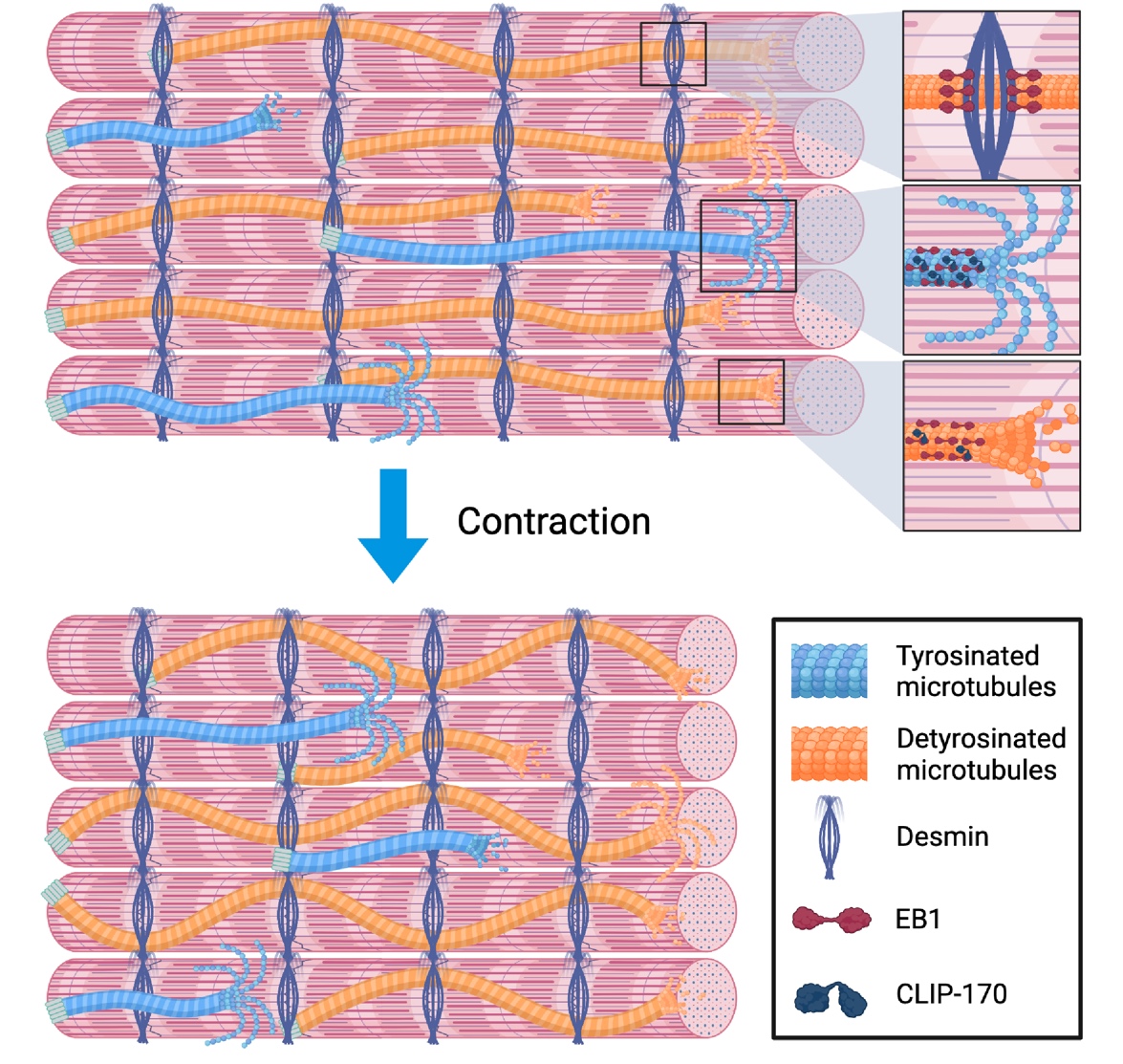
**

**Supplemental Fig. 6** *Cartoon summary of the results: Desmin intermediate filaments and tubulin detyrosination stabilize growing microtubules in the cardiomyocyte*
